# Multiple Electroencephalogram Recordings for Monitoring the Evolution of Neurological Complications during Baclofen Withdrawal Syndrome

**DOI:** 10.1155/2022/4245667

**Published:** 2022-03-07

**Authors:** Maenia Scarpino, Giovanni Lanzo, Cosimo Chelazzi, Antonio Maiorelli, Valentina Bessi, Martina Focardi, Francesco Lolli, Antonello Grippo

**Affiliations:** ^1^Neurophysiology Unit, Careggi University Hospital, Florence, Italy; ^2^Department of Anesthesiology and Intensive Care, Careggi University Hospital, Florence, Italy; ^3^Department of Neurological and Psychiatric Sciences, University of Florence, Italy; ^4^Forensic Medical Sciences, Health Sciences Department, University of Florence, Italy; ^5^Department of Biomedical, Experimental and Clinical Sciences “Mario Serio”, Department of Neuroscience, Italy

## Abstract

Baclofen withdrawal syndrome represents a clinical emergency that can lead to life-threatening complications. It is often a diagnostic challenge because of its nonspecific nature of presentation and degree of symptom overlap with other clinical diseases. Electroencephalography (EEG) might provide important supporting evidence when neurological complications are involved. We present the case of a 55-year-old woman with sudden onset of motor manifestations at the limbs and an altered mental status 24 hours after cessation of intrathecal baclofen administration, following the removal of the pump due to infection, in whom a computed tomography did not show any acute-onset brain injuries, and multiple EEG recordings were performed. The first EEG showed the presence of bilateral sharply contoured waves, in the absence of epileptic discharges and seizures. No correlation between motor manifestations and EEG changes were detected. This EEG pattern was considered to be the expression of an overexcitation of the central nervous system (CNS) due to the loss of baclofen inhibitory effects, excluding an epileptic origin of motor manifestations. Another EEG, performed 24 hours later, showed the presence of triphasic waves with severe generalised slowing, suggesting the presence of encephalopathy. The last EEG, performed 48 hours after the previous recording, when a recovered state of consciousness was already present, showed regression of the triphasic waves and a reorganisation of the background activity. In our case, repeated EEG evaluation allowed monitoring the evolution of acute encephalopathy developed during baclofen withdrawal syndrome, from the initial phase of CNS hyperexcitability, through the phase of metabolic encephalopathy, and to its resolution. This modality allowed for optimising the diagnostic-therapeutic management of the patient during her stay in the intensive care unit.

## 1. Introduction

Baclofen, an agonist for gamma-aminobutyric acid (GABA) receptors [1], is an effective therapeutic mainly for the treatment of spasticity related to spinal cord injuries, other spinal cord diseases, and multiple sclerosis [2]. In recent years, however, as its clinical use has increased—such as for treatment of musculoskeletal pain and muscle spasm—its potential side effects, including life-threatening toxicity and withdrawal syndrome, have become more frequent [3]. Baclofen toxicity and withdrawal syndrome, especially when related to intrathecal administration, can often determine severe multisystem organ failure requiring admission to the intensive care unit (ICU) to administer supportive measures to ensure vital sign stability. Moreover, neurological signs such as seizures [4, 5], encephalopathy [6], and altered mental status are common to both the syndromes [3]. However, while the electroencephalographic (EEG) patterns detected during baclofen toxicity syndrome have been reported widely in the literature [4, 7, 8], the EEG patterns related to the sudden cessation of baclofen administration—leading to withdrawal syndrome—and thus supporting the effective clinical diagnosis of seizures or encephalopathy have not been described.

We present the case of a patient with sudden onset of anxiety and nonspecific motor manifestations at both the lower and upper limbs 24 hours after cessation of intrathecal baclofen administration, following the removal of the pump due to infection, in whom multiple EEG recordings were performed over time and contributed to proper clinical workout of the patient.

## 2. Case Report

A 55-year-old woman with paraplegia, following a spinal cord injury at D4 level, from dorsal meningioma surgery in 2013, started baclofen intrathecal treatment for spasticity in 2017 with a dose of 75 *μ*g/day. She showed normal liver and kidney function markers. She was admitted to an inpatient ward of Careggi Teaching Hospital on 29 October 2021, for the removal of the baclofen pump due to infection. Twenty-four hours after cessation of baclofen administration, the patient showed a sudden onset of anxiety followed by nonspecific motor manifestations at the lower and upper limbs. Therefore, she was started on oral baclofen (25 mg twice a day) alongside the benzodiazepines (diazepam and clonazepam), with only a partial reduction of motor manifestations which were still present at the lower limbs. She underwent brain computed tomography that did not show acute-onset brain injuries even after the administration of contrast medium. The patient was thus admitted to the ICU for proper diagnostic and therapeutic management, undergoing vital sign monitoring, oral administration of baclofen (25 mg three times a day), and intravenous administration of midazolam (0.02-0.05 mg/kg/hour), propofol (1-2 mg/kg/hour), and dexmedetomidina (0.15 *μ*g/kg/hour), however without intubation. A blood gas analysis at admission to the ICU showed a partial pressure of oxygen (pO_2_) of 93 mmHg, a partial pressure of carbon dioxide (pCO_2_) of 38 mmHg, and pH values in the range of normality. A hemodynamic stability was detected during all the ICU stay.

**Figure 1:**
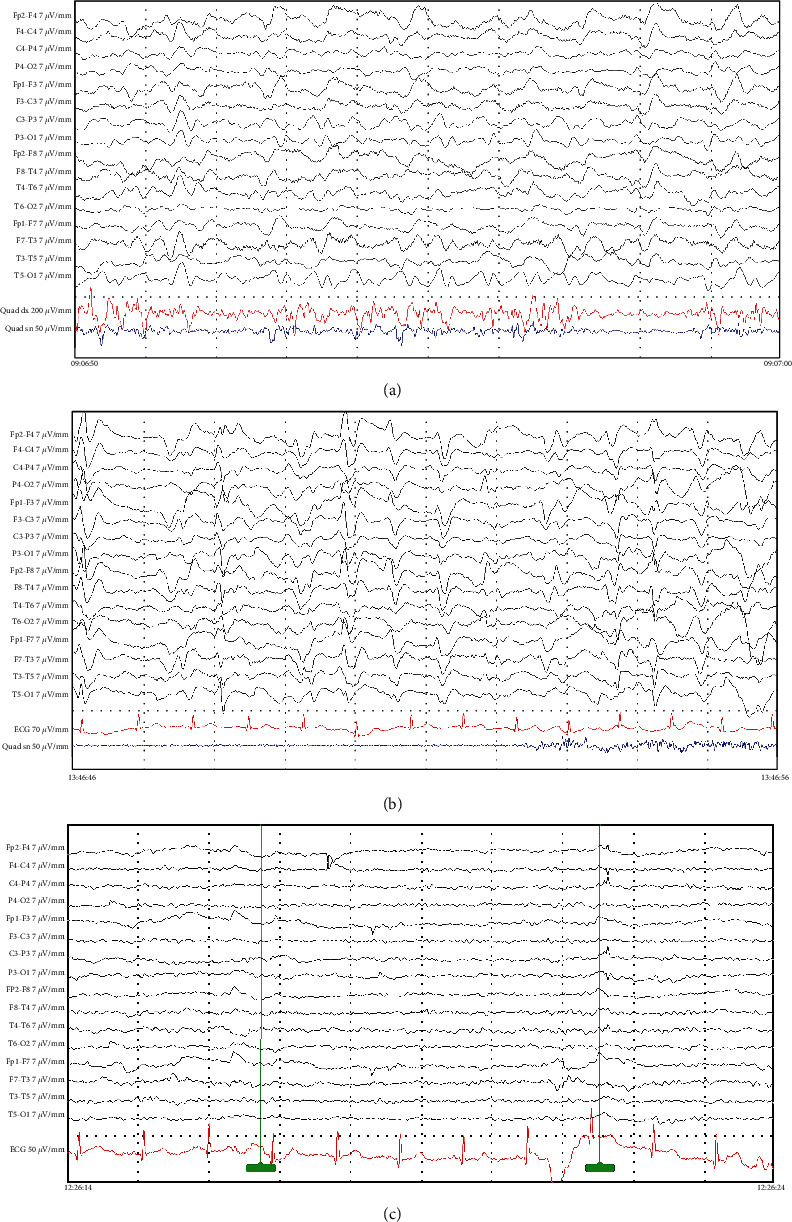
Serial EEG recordings. (a) First day after symptoms onset: presence of abundant and high sharply contoured bilateral waves in the absence of interictal epileptiform waves or discharges. There were no correlations between lower limb motor manifestations and EEG changes. The topography of the background activity was still partially preserved, although a slight slowing was observed. (b) The EEG pattern showed the presence of abundant triphasic waves with severe generalised slowing, suggesting the presence of encephalopathy. The topography of the background activity was no longer detected. (c) EEG, performed about 48 hours after the second recording, showed complete regression of the triphasic waves and restoration of the topography of the background activity, which had only slight slowing.

Based on suspicion of an epileptic origin of the motor manifestations, the patient underwent an EEG about 4 hours after cessation of midazolam administration and 1 hour after cessation of propofol administration. The EEG pattern showed the presence of abundant and high sharply contoured bilateral waves [9] in the absence of epileptic discharges and seizures. In particular, there were no correlations between lower limb motor manifestations and EEG changes. The topography of the background activity was still partially preserved, although a slight slowing was observed (Figure 1(a)). From the clinical point of view, the patient showed nonspecific lower limb motor manifestations but with a partially preserved mental status, occasionally executing simple commands, although there was ideomotor slowing. Treatment with midazolam and propofol, restarted during the EEG recording, resulted in a significant reduction in the above-mentioned EEG anomalies. Because the motor manifestations had a nonepileptic origin, no antiepileptic drugs were administered.

Twenty-four hours later, another EEG was performed some hours after the cessation of intravenous anaesthetic drug administration. The EEG pattern showed the presence of abundant triphasic waves with severe generalised slowing, suggesting the presence of encephalopathy. The topography of the background activity was no longer detected (Figure 1(b)). Clinically, the patient showed less extensive motor manifestations, although they were present at all four limbs, alongside an altered mental status. In this second recording too, no correlations between motor manifestations and EEG changes were detected. Elevation of serum creatine kinase, myoglobin, and potassium was detected, in the absence of the increase of creatinine and sepsis marker levels. Fever was not present. The day after the second EEG, intravenous administration of midazolam and propofol was gradually interrupted in the absence of motor manifestations. Another EEG, performed about 48 hours after the second recording, showed complete regression of the triphasic waves and restoration of the topography of the background activity, which had only slight slowing (Figure 1(c)). Clinically, no motor manifestations were present and the patient showed restoration of consciousness, with only slight ideomotor slowing. The day after the third EEG, the patient was readmitted to the previous inpatient ward for restoration of intrathecal drug delivery as soon as possible, continuing in the meantime oral baclofen administration.

## 3. Discussion

The recent expansion in the use of baclofen to treat spasticity of different causes has been associated with a significant increase in its complications, including toxicity events and withdrawal syndrome, especially regarding intrathecal baclofen administration [3]. In particular, withdrawal syndrome is observed frequently after sudden cessation of intrathecal baclofen administration mainly due to pump malfunction, migration of the intrathecal catheter, or, as in our case, removal of the pump after infection [10, 11]. Withdrawal syndrome represents a clinical emergency related to baclofen use that, if not properly treated, can lead to rhabdomyolysis, autonomic instability, and severe multisystem organ failure associated with life-threatening complications [12, 13]. However, this clinical condition often represents a diagnostic challenge for the clinician because of its nonspecific presentation and degree of symptom overlap with other clinical diseases [14, 15]. Indeed, when it occurs in its severe form, withdrawal syndrome can mimic other clinical conditions including sepsis, neuroleptic malignant syndrome, malignant hyperthermia, or other hypermetabolic states [3]. Hence, prompt recognition and great suspicion are always required, especially when the causes determining the withdrawal syndrome are not evident (i.e., pump malfunction or migration of the intrathecal catheter). Neurological signs during withdrawal syndrome may be related to excessive neurological excitation due to the sudden cessation of the inhibitory action of baclofen in both the central nervous system (CNS) and the peripheral nervous system (PNS) [16, 17], which results in the return of baseline spasticity as well as in the onset of hyperreflexia, tremor, and seizures. However, as seen in our case, it is often challenging to distinguish between motor manifestations of a nonepileptic nature, such as tremor or spasms, and motor myoclonic jerks associated with the presence of seizures. In this clinical context, EEG might be very useful, especially considering that another frequent complication of baclofen withdrawal syndrome is metabolic encephalopathy.

This neurological manifestation may develop as a result of several clinical conditions occurring during baclofen withdrawal syndrome, including elevation in creatinine or in potassium levels and culminating in severe multisystem organ failure. The use of antiepileptic drugs, especially when excessive, might worsen the metabolic encephalopathy and, consequently, exacerbate the altered mental status associated with this clinical condition. Performing EEG could thus provide two advantages: on the one hand, it allows clinicians to distinguish between an epileptic or a nonepileptic origin of any patient's motor manifestations during the course of withdrawal syndrome, allowing improvement of the therapeutic management. On the other hand, it might allow clinicians to identify the possible development of metabolic encephalopathy.

In our case, the first EEG, performed at an early stage after the onset of the motor manifestations of the limbs, allowed us to determine a nonepileptic origin of the clinical signs, thus avoiding starting inappropriate antiepileptic therapy. Indeed, the presence of the abundant high sharply contoured waves, in the absence of epileptic discharges and seizures, could be considered the expression of CNS overexcitation due to the loss of the inhibitory effects of baclofen.

Avoiding the administration of inappropriate antiepileptic treatment was crucial in our patient, in whom encephalopathy developed the day after the onset of the first neurological symptoms. In the present case, in which withdrawal syndrome occurred because the pump had been removed due to infection, the second EEG pattern characterised by severe generalised slowing and abundant triphasic waves, signs of encephalopathy, could not distinguish between a septic or a metabolic origin of the neurological manifestation. However, the prompt (<48 hours) resolution of the parossistic triphasic discharges on EEG, which also showed reorganisation of the topography and only minimal slowing of the frequency of the background activity, in the absence of fever and elevated biochemical markers of sepsis (such as increased white blood cells) guided us to determine the encephalopathy had a metabolic origin related to baclofen withdrawal syndrome, rather than a septic cause.

## 4. Conclusion

Withdrawal syndrome represents a concerning complication of baclofen therapy. Prompt recognition and great clinical suspicion are mandatory because if not treated, withdrawal syndrome might lead to multisystem organ failure and even death within few days. In our case, baclofen withdrawal syndrome therapy comprised reinitiation of baclofen and the use of drugs that mimic the mechanism of action of baclofen, such as benzodiazepines and propofol, besides hydration to prevent and treat rhabdomyolysis, vital sign monitoring, and cardiopulmonary support [5, 12]. In the course of baclofen withdrawal syndrome, as well as in the course of baclofen toxicity, instrumental tests, such as EEG, might provide important supporting evidence besides clinical signs and symptoms that often could be nonspecific and overlap with other diseases, especially when the cause of symptoms is not well understood (i.e., pump malfunction or migration of the intrathecal catheter). EEG, a bed-side, inexpensive, and rapid test, provides a functional evaluation of the nervous system, complementing and integrating neuroradiological techniques, and it is very useful when neuroimaging shows no pathological findings [18, 19]. Continuous EEG monitoring should be used in critically ill patients admitted to intensive care due to presumed CNS pathology providing an important real-time information in detecting subclinical seizures and nonconvulsive status epilepticus or to monitor evolution of the acute encephalopathy. When continuous EEG monitoring is not possible, at least repeated daily, EEG evaluation allowed monitoring the evolution of the acute encephalopathy, due to baclofen withdrawal syndrome, from an initial phase of CNS hyperexcitability, through the phase of metabolic encephalopathy, and to its resolution, optimising the diagnostic-therapeutic management of the patient during ICU stay.

## Data Availability

No data were used to support this study.
